# Survival Outcomes and Prognostic Factors in Metastatic Unresectable Appendiceal Adenocarcinoma Treated with Palliative Systemic Chemotherapy: A 10-Year Retrospective Analysis from Australia

**DOI:** 10.3390/cancers17203297

**Published:** 2025-10-11

**Authors:** Jirapat Wonglhow, Hui-Li Wong, Michael Michael, Alexander Heriot, Glen Guerra, Catherine Mitchell, Jeanne Tie

**Affiliations:** 1 Division of Medical Oncology, Department of Internal Medicine, Faculty of Medicine, Prince of Songkla University, Songkhla 90110, Thailand; 2 Department of Medical Oncology, Peter MacCallum Cancer Centre, Melbourne, VIC 3000, Australia; huili.wong@petermac.org (H.-L.W.); michael.michael@petermac.org (M.M.); jeanne.tie@petermac.org (J.T.); 3 Sir Peter MacCallum Department of Oncology, University of Melbourne, Melbourne, VIC 3000, Australia; catherine.mitchell@petermac.org; 4 Division of Cancer Surgery, Peter MacCallum Cancer Centre, Melbourne, VIC 3000, Australia; alexander.heriot@petermac.org (A.H.); glen.guerra@petermac.org (G.G.); 5 Department of Pathology, Peter MacCallum Cancer Centre, Melbourne, VIC 3000, Australia

**Keywords:** appendiceal cancer, adenocarcinoma, metastasis, palliative chemotherapy, survival, efficacy

## Abstract

Background: Appendiceal adenocarcinoma is a rare malignancy, and data guiding its systemic treatment in metastatic settings are limited. This study aimed to determine the clinical outcomes, treatment efficacy, biomarkers, and prognostic factors in patients with metastatic or unresectable appendiceal adenocarcinoma receiving palliative chemotherapy. Methods: We retrospectively reviewed patients with metastatic appendiceal adenocarcinoma who received first-line palliative systemic chemotherapy at the Peter MacCallum Cancer Centre between January 2015 and December 2024. Results: Of the 40 patients included, fluoropyrimidine-based doublet regimens were most commonly used (82.5%) in first-line setting, achieving an objective response rate of 39.4%. Median overall survival (OS) was 21.6 months, and median first-line progression-free survival (PFS) was 8.9 months. 22 patients (55.0%) received second-line treatment. Median OS and PFS were 21.6 and 8.9 months, respectively, among patients treated with oxaliplatin-based doublet regimens, and 66.4 and 10.8 months, respectively, among those treated with irinotecan-based doublet regimens. Molecular biomarker testing was performed in 35 patients (87.5%). *KRAS* and *NRAS* mutations were identified in 68.6% and 2.9% of tested patients, respectively. Factors associated with poorer OS included male sex, elevated carcinoembryonic antigen levels, and overweight status. Bevacizumab use was not clearly associated with survival. Conclusions: Palliative systemic chemotherapy, particularly fluoropyrimidine-based doublet regimens, appears to be a reasonable and effective treatment option for patients with advanced appendiceal adenocarcinoma. Although this study was underpowered for formal comparison, the numerically longer OS and PFS of irinotecan-based regimens are hypothesis-generating and support further prospective evaluation. Molecular profiling emphasizes the need for personalized targeted therapeutic strategies. The identified prognostic factors may help guide risk stratification and patient counseling for treatment planning.

## Simple Summary

Appendiceal adenocarcinoma is a rare cancer, and there is limited evidence to guide systemic treatment for metastatic or unresectable cases. This study evaluated real-world outcomes in patients with advanced appendiceal adenocarcinoma who received palliative chemotherapy. The results support fluoropyrimidine-based doublet chemotherapy as an appropriate and preferred option for patients with high disease burden and aggressive tumor features. Single-agent fluoropyrimidine therapy may be a reasonable alternative for frail patients with less aggressive disease. A high incidence of *KRAS* mutations was observed, which may affect the use of targeted therapies. These findings highlight the need for treatment strategies tailored specifically to appendiceal cancer and suggest that molecular testing may help guide future therapy. This study offers valuable insights to improve care for patients with this rare and understudied disease.

## 1. Introduction

Epithelial neoplasms of the appendix exhibit a wide spectrum of biological behaviors, ranging from indolent lesions such as low-grade appendiceal mucinous neoplasms to more aggressive forms such as high-grade adenocarcinomas [1]. Despite this heterogeneity, malignant tumors arising from the appendix remain exceedingly uncommon, comprising merely 0.4% of all gastrointestinal tract malignancies according to data from the Surveillance, Epidemiology, and End Results (SEER) database [2]. Appendiceal cancer is an exceptionally rare malignancy with an estimated annual incidence of only 1–2 cases per million people [3,4].

Although the majority (approximately 75%) of appendiceal mucinous neoplasms are noninvasive and exhibit slow progression, allowing for prolonged survival even without specialized treatment, a subset demonstrates aggressive features with rapid progression and can be fatal within 1–2 years of diagnosis [5]. Appendiceal adenocarcinomas are histologically classified into mucinous, nonmucinous, and signet ring cell subtypes [6,7]. Notably, patients with stage IV nonmucinous tumors tend to have worse five-year overall survival (OS) rates than those with mucinous subtypes, particularly low-grade variants [8,9,10]. These survival discrepancies point to possible underlying biological differences between mucinous and nonmucinous adenocarcinomas [11,12,13]; however, data are currently limited to assess the treatment effects across these histologic subtypes.

The existing literature on appendiceal adenocarcinoma largely stems from surgical series with a primary focus on operative interventions and postoperative outcomes [9,14,15,16,17]. For patients with mucinous histology and peritoneal dissemination, particularly in low-grade tumors, cytoreductive surgery (CRS) combined with hyperthermic intraperitoneal chemotherapy (HIPEC) has emerged as the preferred treatment approach [2,18,19,20,21]. Conversely, the role of systemic chemotherapy has been extensively explored in the neoadjuvant and adjuvant contexts, typically in surgically treated cohorts [22,23,24,25,26]. In patients with inoperable or metastatic disease, systemic palliative chemotherapy remains the mainstay of treatment and is often extrapolated from colorectal cancer (CRC) protocols [27,28,29]. Fluoropyrimidine-based doublet regimens are commonly used; however, no well-designed randomized controlled trials have been conducted in this rare population [9,30,31,32]. Consequently, data regarding the efficacy of systemic chemotherapy for appendiceal adenocarcinomas are limited.

Given the rarity of appendiceal adenocarcinoma and the scarcity of data on palliative chemotherapy, treatment outcomes in this setting remain poorly defined. This study aimed to address this gap by evaluating OS, progression-free survival (PFS), objective response rate (ORR), molecular, and prognostic factors in real-world patients with metastatic appendiceal adenocarcinoma receiving palliative systemic chemotherapy from a single institution.

## 2. Materials and Methods

### 2.1. Study Participants and Procedures

We performed a retrospective chart review of patients diagnosed with appendiceal adenocarcinoma at the Peter MacCallum Cancer Centre in Melbourne, Australia, between January 2015 and December 2024. Eligible participants met the following criteria: (1) histopathologically confirmed appendiceal adenocarcinoma, or high-grade appendiceal mucinous neoplasms; (2) metastatic disease classified as cTxNxM1 based on the American Joint Committee on Cancer 8th edition staging system; (3) receipt of at least one cycle of first-line palliative systemic chemotherapy; and (4) age 18 years or older. The exclusion criteria comprised patients treated solely with surgery or HIPEC without systemic chemotherapy, those who underwent adjuvant systemic chemotherapy following CRS with HIPEC and had no residual disease, or individuals diagnosed with low-grade appendiceal mucinous neoplasm.

Patient data were retrieved from the institution’s EPIC electronic medical records. The extracted variables included demographic and clinical characteristics such as age, sex, body mass index, Eastern Cooperative Oncology Group (ECOG) performance status, baseline laboratory values, tumor histology, TNM staging, metastatic sites, and relevant molecular profiles. Treatment details including chemotherapy regimens, prior interventions, and subsequent therapies were also recorded.

This study received approval from the Human Research Ethics Committee and Governance Office of the Peter MacCallum Cancer Centre (HREC: QA/117546/PMCC). Due to the retrospective design of this study, as per institutional approval, the requirement for written informed consent was waived. All patient identifiers were anonymized to ensure confidentiality and data protection.

### 2.2. Outcome Measurements

The primary endpoint of this study was OS of patients with metastatic appendiceal adenocarcinoma who received first-line palliative chemotherapy. Secondary endpoints included PFS, ORR to first-line therapy, and identification of prognostic factors including molecular markers for OS.

OS was calculated from the date of chemotherapy initiation until death from any cause. PFS was defined as the interval from the start of chemotherapy to either radiologically confirmed disease progression or death, whichever occurred first. Treatment response was assessed every 2–3 months using chest and abdominal computed tomography.

Tumor response was classified into four categories: complete response (CR), defined as the disappearance of all radiographic and clinical evidence of disease; partial response (PR), indicating any measurable reduction in tumor burden without clinical progression; stable disease (SD), reflecting minimal or no significant change in tumor size or clinical symptoms; and progressive disease (PD), indicating clear tumor progression, new lesions, or clinical deterioration. Patients with either CR or PR were considered to have an objective response to treatment, which contributed to the ORR.

### 2.3. Statistical Analysis

Continuous variables were summarized using either median and interquartile range or mean and standard deviation based on their distribution. Categorical variables are presented as counts and percentages. Survival outcomes were estimated using the Kaplan–Meier method, and differences between groups were assessed using the log-rank test. Univariate and multivariate Cox proportional hazards regression analyses were performed to identify prognostic factors. All statistical analyses were conducted using the R software version 4.3.1 (R Foundation, Vienna, Austria). Two-sided *p*-values less than 0.05 were considered significant.

## 3. Results

### 3.1. Baseline Characteristics

This study included 40 patients diagnosed between 2015 and 2024 who fulfill the selection criteria. Their baseline characteristics are summarized in Table 1. Median age was. 56 years, 60% of patients were female, majority had an ECOG performance status of 0 or 1. Sixty percent of the patients were diagnosed with de novo metastatic disease, most of whom had metastases limited to a single site. All patients exhibited peritoneal involvement. The most common histological subtypes were adenocarcinoma and mucinous adenocarcinoma, with approximately one-third of patients exhibiting signet ring cell features. More than half of the patients had normal carcinoembryonic antigen (CEA) and carbohydrate antigen 19-9 (CA19-9) levels. Among patients who underwent fluorodeoxyglucose (FDG) positron emission tomography (PET), one-fourth had non-FDG-avid disease. Within this latter subgroup, 50% were mucinous subtype, 40% adenocarcinoma, and 10% goblet cell adenocarcinoma.

### 3.2. Molecular Biomarker Assessment

Molecular biomarker data, including *KRAS*, *NRAS*, *BRAF*, and mismatch repair (MMR) status, were unavailable for five patients. Among the 35 patients with evaluable data, all were tested using targeted colorectal cancer gene panels. *KRAS* and *NRAS* mutations were identified in 68.6% and 2.9%, respectively. *BRAF* mutations or deficient MMR were not observed. Regarding human epidermal growth factor receptor 2 (HER2) testing, only 13 patients were evaluated. All tested negative according to the standard HER2 positivity criteria; however, approximately half of those tested were HER2-low, defined as immunohistochemistry 1+ or 2+ with negative fluorescent in situ hybridization. The detailed molecular profiles are summarized in Table 2.

### 3.3. Treatment Information

Details of the first- and second-line treatments are summarized in Table 3. Approximately half of the patients received only one line of palliative systemic therapy. Most patients received doublet chemotherapy with 5-fluorouracil (5-FU) plus leucovorin and oxaliplatin (FOLFOX) followed by 5-FU plus leucovorin and irinotecan (FOLFIRI). Bevacizumab was added to chemotherapy in 20% of the cases.

First-line treatment was primarily discontinued due to disease progression. Other reasons included undergoing CRS with HIPEC or completing six months of therapy followed by a planned treatment break. Approximately half of the cohort proceeded to second-line treatment. Among the 18 patients who did not proceed to second-line therapy, the main reasons were rapid clinical deterioration to ECOG performance status 3 from progressive disease (50%) and gut obstruction not suitable for surgery (22%). Less common reasons included poor tolerance to chemotherapy, patient preference, or development of sepsis, while one patient was still receiving first-line treatment at the data cutoff.

In the second-line setting, 27.3% of patients received bevacizumab, and 13.6% received cetuximab in combination with chemotherapy. FOLFIRI was the most commonly used second-line regimen, followed by FOLFOX. Additional information on subsequent treatments is provided in Table S1. Only 3 (7.5%) of patients were enrolled on early phase clinical trials.

### 3.4. OS

**Figure 1:**
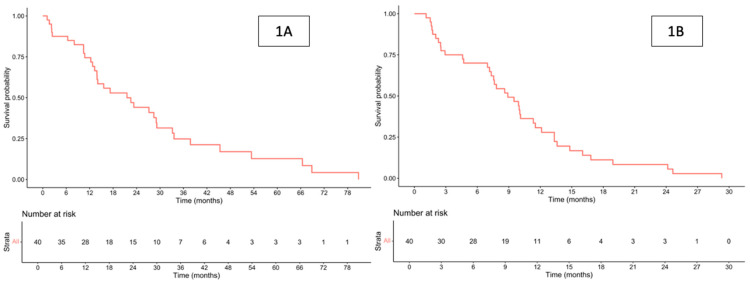
Overall survival (**A**) and progression-free survival (**B**) for all patients.

**Figure 2:**
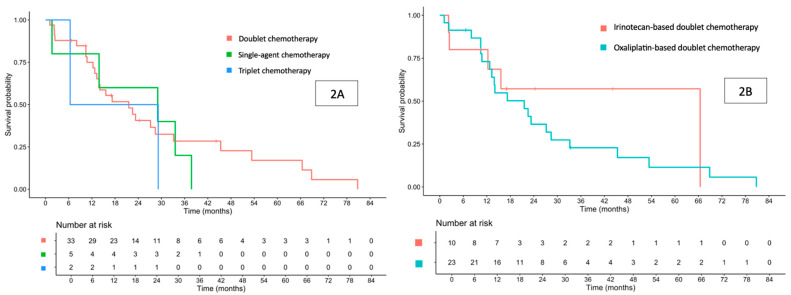
Overall survival in patients treated with first-line palliative doublet, single-agent, or triplet chemotherapy (**A**), and overall survival in patients treated with first-line palliative oxaliplatin-based or irinotecan-based doublet chemotherapy (**B**).

The median follow-up duration was 17.1 (range, 1.1–80.8) months. The median OS for the entire cohort was 21.6 (95% confidence interval [CI] 13.9–33.2) months (Figure 1A). When stratified by first-line palliative chemotherapy regimens, the median OS was 29.0 months for single-agent therapy, 21.6 months for doublet therapy, and 17.8 months for triplet therapy (Figure 2A). Among patients who received doublet chemotherapy, those treated with oxaliplatin-based regimens had a median OS of 21.6 months, while 66.4 months for those receiving irinotecan-based regimens (Figure 2B).

**Figure 3:**
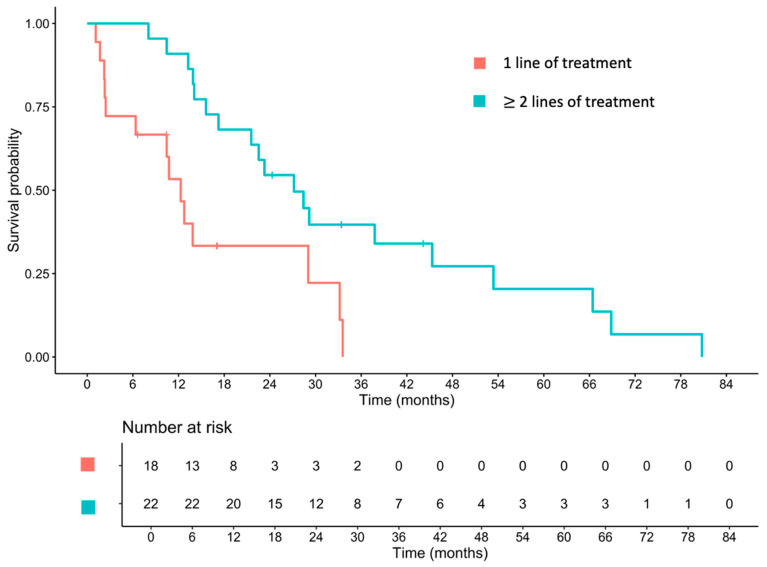
Overall survival of patients who received only one line of systemic therapy and those who received at least two lines of systemic therapy.

Patients who received second-line treatment had a significantly longer median OS than those who received only first-line therapy (27.2 vs. 12.3 months; HR 0.33, 95% CI 0.15–0.71, *p* = 0.005; Figure 3).

### 3.5. PFS

**Figure 4:**
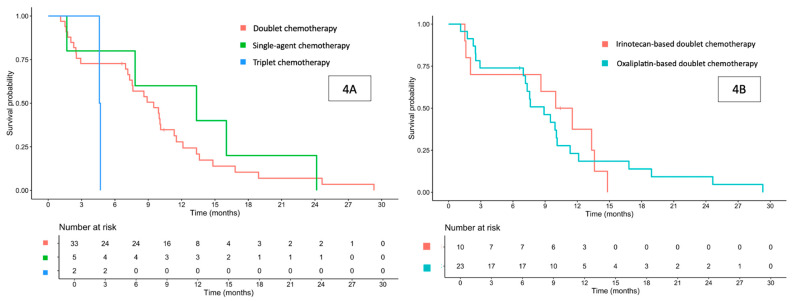
Progression-free survival in patients treated with first-line palliative doublet, single-agent, or triplet chemotherapy (**A**), and progression-free survival in patients treated with first-line palliative oxaliplatin-based or irinotecan-based doublet chemotherapy (**B**).

The median PFS for the entire cohort was 8.9 (95% CI 7.33–11.5) months (Figure 1B). When stratified according to the type of first-line palliative chemotherapy, the median PFS was 13.3 months for single-agent therapy, 9.5 months for doublet chemotherapy, and 4.7 months for triplet regimens (Figure 4A).

Among patients who received doublet chemotherapy, those treated with oxaliplatin-based regimens had a median PFS of 8.9 months, while 10.8 months for those receiving irinotecan-based regimens (Figure 4B). The addition of bevacizumab to doublet chemotherapy yielded a median PFS of 9.49 months, while 8.61 months without bevacizumab.

When evaluating individual regimens, the median PFS times were as follows: FOLFOX (7.6 months), capecitabine plus oxaliplatin (CAPOX; 10.1 months), FOLFIRI (10.8 months), 5-FU plus leucovorin, oxaliplatin, and irinotecan (FOLFOXIRI; 4.7 months), capecitabine (13.3 months), and 5-FU (12.9 months).

### 3.6. Response Rates

As shown in Table 4, the ORR was 39.4% in patients who received doublet chemotherapy. No objective response was observed in patients treated with either single-agent or triplet regimens.

Among those receiving doublet chemotherapy, the ORRs were 43.5% for oxaliplatin-based regimens and 30.0% for irinotecan-based regimens (Table S2). The ORR was similar in patients who received bevacizumab in combination with doublet chemotherapy (41.2%) and those who did not receive bevacizumab (37.5%; Table S3). Response rates for adenocarcinoma and mucinous adenocarcinoma subtypes are presented in Table S4.

### 3.7. Prognostic Factors

To identify factors associated with OS, univariate Cox regression analysis was performed on various clinical parameters. Variables with potential significance (*p* < 0.15) were further assessed using multivariate Cox regression (Table 5). In the univariate analysis, male sex, non-normal BMI (underweight or overweight), elevated baseline CEA levels ( ≥ 5 U/mL), poor tumor differentiation, FDG-PET avidity, and synchronous metastatic disease were associated with worse OS. However, in the multivariate analysis, only male sex, overweight status, and elevated CEA levels remained independently associated with worse prognosis. 

## 4. Discussion

Only limited evidence exists regarding the role of palliative systemic chemotherapy in patients with unresectable or metastatic appendiceal adenocarcinoma. Due to the rarity of this disease, no well-designed randomized controlled trials have been conducted to determine the optimal chemotherapeutic regimen. This study evaluated patients with unresectable metastatic appendiceal adenocarcinoma who received first-line palliative chemotherapy. The median OS and PFS were 21.6 months and 8.9 months, respectively. Most patients (82.5%) were treated with doublet chemotherapy, followed by single-agent therapy (12.5%) and triplet chemotherapy (5%) with or without the addition of bevacizumab. Fluoropyrimidine-based doublet chemotherapy achieved an ORR of 39.4% and a disease control rate (ORR and SD) of 72.7%, with a median PFS of 9.5 months and a median OS of 21.6 months.

The median PFS for patients treated with fluoropyrimidine-based doublet chemotherapy regimens from our study is consistent with outcomes reported by previous studies [9,29,30,31]. However, this PFS is shorter than the median of 14.4 months reported in a study from the United States [28], noted that their cohort was younger, and 65% had undergone debulking surgery. Other doublet regimens, including taxane-based chemotherapy, demonstrated a median PFS of 7.4 months in a small cohort of 13 patients [33]. Notably, most previous studies have reported heterogeneous patient populations, histological subtypes, disease stages, and treatment regimens, making comparisons difficult.

In addition to doublet regimens, our study also evaluated outcomes from single-agent and triplet chemotherapies, yielding median PFS values of 13.3 and 4.7 months, respectively. However, these results should be interpreted with caution because of the small sample size and selection biases, which may not fully represent overall efficacy. Patients receiving single-agent chemotherapy tended to be older, with less aggressive disease features characterized by lower rates of poor differentiation, signet ring cell features, and FDG avidity on PET scans, fewer metastatic sites, and a higher proportion of mucinous adenocarcinoma or high-grade appendiceal mucinous neoplasms, whereas those treated with triplet regimens were generally younger and fitter, but with more aggressive tumor biology, including poor differentiation, signet ring cells, multiple metastatic sites, and universally positive FDG PET scans. Although the single-agent group appeared to have a longer median PFS than the doublet group, this difference was not significant and likely reflected a selection bias. Overall, our findings support the use of fluoropyrimidine-based doublet chemotherapy as an appropriate treatment option for unresectable appendiceal adenocarcinoma. Single-agent fluoropyrimidine therapy may be a reasonable alternative for older or frail patients with less aggressive disease biology who are unsuitable for combination regimens.

Regarding OS, the median OS in our cohort was 21.6 months. Although the single-agent group appeared to have a longer median OS numerically, this finding was likely skewed by the small sample size (*n* = 5) and more indolent biology. Survival outcomes are influenced not only by the treatment type but also by patient risk factors, biomarkers, and subsequent therapies. Most publications report a median OS of 20–28 months in cohorts primarily treated with fluoropyrimidine-based doublet regimens, whereas taxane-based chemotherapy has been associated with shorter OS (median 8.8 months) [9,28,29,30,31,33]. Our analysis also demonstrated numerically longer median OS and PFS in the irinotecan group. Although this study was underpowered for a formal comparison. These findings are hypothesis-generating and support prospective studies to evaluate irinotecan-based doublets as an alternative first-line strategy for metastatic appendiceal adenocarcinoma. Notably, patients who received only one line of treatment had significantly poorer survival than those receiving multiple lines. However, this apparent benefit should be interpreted with caution, as patients with indolent disease and good performance status are more likely to proceed to subsequent therapy, whereas those with aggressive biology often deteriorate rapidly or develop complications such as bowel obstruction that preclude further treatment.

Regarding the response rate, we observed an ORR of 39.4%. Direct comparisons with previous reports are challenging because of the wide variability in ORR values reported ranging from 20% to 50% [9,28,30,31,32]. This variability reflects the heterogeneous chemotherapy regimens used, some with bevacizumab or cetuximab and others without, often reported collectively without regimen-specific details. Another important factor is the difficulty of response assessment in patients with peritoneal disease, where lesions are often not measurable according to the Response Evaluation Criteria in Solid Tumors (RECIST). In our study, as in several others, response evaluation relied on combined radiological and clinical assessments rather than strict RECIST criteria, which may contribute to inconsistencies across studies. Nonetheless, both prior studies and ours suggest that the ORR may be influenced by tumor histology. Studies focusing primarily on mucinous adenocarcinoma reported lower ORRs of approximately 20–24% [29,31], whereas studies that predominantly included nonmucinous histology found higher ORRs of 40–50% [9,28,30]. Furthermore, some reports indicated no objective responses among patients with low-grade or well-differentiated mucinous adenocarcinoma, suggesting limited chemotherapy benefits in this subgroup [8,34,35]. Consistent with these findings, we found no ORR in patients treated with single-agent chemotherapy. Collectively, doublet chemotherapy in the first-line setting yielded ORRs ranging from 20% to 50%, with a tendency toward higher response rates in nonmucinous or high-grade mucinous adenocarcinomas. Therefore, doublet chemotherapy remains the preferred treatment option for patients with high disease burden and aggressive tumor features.

The addition of anti-vascular endothelial growth factor monoclonal antibodies such as bevacizumab to chemotherapy has demonstrated clinical benefits for CRC. However, owing to the rarity of appendiceal adenocarcinoma, strong evidence supporting this approach in appendiceal cancer is lacking. Although a previous study suggested that bevacizumab improves both OS and PFS [32], our was relatively small, and therefore we were not able to draw definitive conclusions regarding its benefit. Thus, the role of bevacizumab in this context remains unclear. Currently, the evidence is insufficient to strongly recommend the routine addition of bevacizumab as first-line chemotherapy for appendiceal adenocarcinoma, making it acceptable as a systemic chemotherapy alone.

The role of biological therapies commonly used for advanced CRC, such as anti-epidermal growth factor receptor (EGFR) antibodies, remains unclear in appendiceal cancer. *KRAS* mutations, which are well-established predictive biomarkers of resistance to the anti-EGFR monoclonal antibodies cetuximab and panitumumab in CRC [36], are known to play a role in mucinous cancers of various sites, including the appendix [37,38,39]. Despite the limited sample size, our study found a high incidence of *KRAS* mutations (68.6%), which restricts the utility of anti-EGFR therapies. This finding is consistent with other reports [31] that documented *KRAS* mutations in up to 84% of cases. The *KRAS* mutation rate in appendiceal cancer is higher than that typically observed in CRC, approximately 40% [40]. The predominant *KRAS* variants identified in our cohort were G12D, G12A, and G12V, with no *KRAS* G12C mutations detected. This is notable because *KRAS* G12C inhibitors such as sotorasib and adagrasib have recently been approved as tumor-agnostic therapies for cancers harboring this mutation [41,42]. Beyond G12C, novel therapeutic strategies are rapidly evolving. Both KRAS G12D–specific inhibitors and pan-KRAS or pan-RAS inhibitors are entering clinical evaluation and may represent potential new therapeutic opportunities [43,44]. Such developments could be particularly relevant for appendiceal adenocarcinoma, given the predominance of non-G12C variants in this disease.

In addition to *KRAS*, we observed *NRAS* mutations in 2.9% of the patients, further limiting the use of anti-EGFR antibodies. No *BRAF* mutations or mismatch repair deficiencies were detected in our cohort. HER2 expression was generally negative among the tested patients, although approximately half of this population exhibited HER2-low status. Although no targeted HER2 therapies are currently approved for HER2-low CRC or appendiceal cancers, agents such as trastuzumab deruxtecan have demonstrated clinical benefits in HER2-low breast cancer [45], suggesting potential future avenues for therapy. Beyond these alterations, *GNAS* mutations appear to be a distinct molecular feature of appendiceal adenocarcinoma. They are strongly enriched in appendiceal compared to colorectal cancer and have been linked to poor chemotherapy response and shorter survival [13,46,47]. These findings highlight the unique tumor biology of appendiceal cancer, supporting its consideration as a separate disease entity. Future molecular profiling, including *GNAS* status, may help identify novel therapeutic targets for this rare malignancy.

In our analysis, male sex, overweight status, and elevated baseline CEA were independent predictors of worse OS, consistent with prior studies [48,49,50]. Appendiceal tumors, particularly those with mucinous histology, are typically characterized by low or absent FDG uptake, leading to potentially false negative results on PET imaging [51]. Although FDG-PET avidity did not reach statistical significance in the multivariate model, it showed a trend toward worse survival (*p* = 0.054). Despite its limitations in staging and response assessment, FDG PET may still provide useful prognostic information in selected patients and warrants further investigation.

This study provided a comprehensive real-world evaluation of patients with metastatic appendiceal adenocarcinoma treated with palliative chemotherapy, including treatment efficacy, survival outcomes, biological assessments, and prognosis. Given the rarity of appendiceal cancer, prospective studies are challenging to conduct. Our retrospective single-center design is limited by a relatively small sample size, restricting the statistical power of the analyses, especially in subset comparisons by treatment regimens. We acknowledge the inherent selection bias related to treatment allocation. Another notable challenge in studying this malignancy is the standardized evaluation of the tumor response. Traditional radiological criteria, such as the RECIST criteria, are often difficult to apply because of imaging characteristics specific to appendiceal tumors. Consequently, the response assessments in this study were based on both clinical and radiological judgments from a multidisciplinary team meeting, introducing a degree of subjectivity. All data were collected from a single institution, and evaluations were performed by a limited group of clinicians, which helped minimize interobserver variability and added consistency to the response assessments, despite the absence of uniformly applicable imaging criteria. In addition, given the retrospective nature of this study, histopathological samples were not re-reviewed. Some pathological details, such as the percentage of signet-ring cell involvement, were inconsistently reported and precluded accurate classification of signet-ring cell carcinoma as a separate subgroup, which may have limited further pathological subgroup analyses.

## 5. Conclusions

Given the rarity of appendiceal adenocarcinoma and the limited data on its treatment, our study provides valuable evidence supporting the use of palliative chemotherapy in advanced cases. Despite its limitations, this study offers two key clinical insights. First, fluoropyrimidine-based systemic chemotherapy is a reasonable and valid treatment option for patients with advanced appendiceal adenocarcinoma. However, the optimal chemotherapeutic regimen remains unclear. Further prospective multicenter studies exploring the benefit of first-line irinotecan-based doublet regimens are warranted. Nevertheless, the benefits of biological agents, particularly those of bevacizumab, remain controversial. Notably, *KRAS* mutations are frequently observed and may represent a therapeutic target with emerging KRAS or RAS targeted agents. Although no HER2-positive cases were detected, approximately half of the patients exhibited HER2-low status. These findings highlight the need for further research involving biomarker testing and biological therapies to expand the treatment options. Second, the identified prognostic factors can assist clinicians in counseling patients regarding the expected outcomes.

## Figures and Tables

**Table 1:** Baseline characteristics.

	Doublet Regimen (*n* = 33)	Single-Agent Regimen (*n* = 5)	Triplet Regimen (*n* = 2)	Total (*n* = 40)
Female, *n* (%)	22 (66.7)	2 (40.0)	0 (0)	24 (60.0)
Age, median, years (IQR)	56.0 (43.0, 68.4)	70.5 (49.5, 74.2)	46.3 (41.5, 51.1)	55.9 (44.6, 68.5)
Age ≥ 65 years, *n* (%)	12 (36.4)	3 (60.0)	0 (0)	15 (37.5)
BMI kg/m^2^, *n* (%)				
<18.5	2 (6.1)	0 (0)	0 (0)	2 (5.0)
18.5–24.9	9 (27.3)	2 (40.0)	1 (50.0)	12 (30.0)
≥25.0	22 (66.7)	3 (60.0)	1 (50.0)	26 (65.0)
ECOG PS, *n* (%)				
0	22 (66.7)	1 (20.0)	2 (100.0)	25 (62.5)
1	11 (33.3)	3 (60.0)	0 (0)	14 (35.0)
2	0 (0)	1 (20.0)	0 (0)	1 (2.5)
Metastatic type, *n* (%)				
Synchronous	20 (60.6)	2 (40.0)	2 (100.0)	24 (60.0)
Metachronous	13 (39.4)	3 (60.0)	0 (0)	16 (40.0)
Number of metastasis sites, *n* (%)				
1	21 (63.6)	4 (80.0)	1 (50.0)	26 (65.0)
≥2	12 (36.4)	1 (20.0)	1 (50.0)	14 (35.0)
Site of metastasis, *n* (%)				
Peritoneum	33 (100.0)	5 (100.0)	2 (100.0)	40 (100.0)
Ovary	7 (21.2)	0 (0)	0 (0)	7 (17.5)
Liver	4 (12.1)	1 (20.0)	0 (0)	5 (12.5)
Lung	1 (3.0)	1 (20.0)	0 (0)	2 (5.0)
Pleura	1 (3.0)	0 (0)	1 (50.0)	2 (5.0)
Distant lymph node	2 (6.1)	0 (0)	0 (0)	2 (5.0)
Adrenal	0 (0)	1 (20.0)	0 (0)	1 (2.5)
Bone	1 (3.0)	0 (0)	0 (0)	1 (2.5)
Histology, *n* (%)				
Adenocarcinoma	17 (51.5)	2 (40.0)	0 (0)	19 (47.5)
Mucinous adenocarcinoma	14 (42.4)	2 (40.0)	2 (100.0)	18 (46.5)
Goblet cell adenocarcinoma	2 (6.1)	0 (0)	0 (0)	2 (5.0)
HAMN	0 (0)	1 (20.0)	0 (0)	1 (2.5)
Differentiation, *n* (%)		*		*
Moderately	17 (51.5)	3 (75.0)	1 (50.0)	22 (53.8)
Poorly	16 (48.5)	1 (25.0)	1 (50.0)	18 (46.2)
Signet ring cell feature, *n* (%)	11 (33.3)	1 (20.0)	1 (50.0)	13 (32.5)
FDG PET scan, *n* (%)				
Avid	24 (72.7)	2 (40.0)	2 (100.0)	28 (70.0)
Non-avid	9 (27.3)	1 (20.0)	0 (0)	10 (25.0)
Not performed	0 (0)	2 (40.0)	0 (0)	2 (5.0)
Baseline laboratory values				
Hemoglobin, g/dL (SD)	12.8 (1.7)	12.5 (1.9)	11.8 (0.2)	12.7 (1.7)
Albumin, g/dL (IQR)	3.7 (3.4, 3.9)	3.6 (3.1, 3.6)	2.8 (2.8, 2.8)	3.7 (3.3, 3.9)
CEA, ng/mL (IQR)	4.2 (1.7, 7.0)	56.3 (22.7, 90.8)	83.0 (45.9, 120)	37.67 (77.72)
CEA ≥ 5 ng/mL, *n* (%)	12/31 (38.7)	4/4 (100.0)	2/2 (100.0)	18/37 (48.6)
CA19-9, U/mL (SD)	198.5 (320.7)	206.4 (102.3)	22.0	206.4 (413.9)
CA19-9 ≥ 37 U/mL, *n* (%)	13/26 (50.0)	2/3 (66.7)	0/1 (0)	15/30 (50.0)
Previous treatment, *n* (%)				
Primary tumor resection	22 (66.7)	3 (60.0)	0 (0)	25 (62.5)
CRS/HIPEC	9 (27.3)	2 (40.0)	0 (0)	11 (27.5)

* excluding HAMN. BMI, body mass index; ECOG PS, Eastern Cooperative Oncology Group performance status; HAMN, high-grade appendiceal mucinous neoplasm; FDG, fluorodeoxyglucose; PET, positron emission tomography; CEA, carcinoembryonic antigen; CA19-9, carbohydrate antigen 19-9; CRS, cytoreductive surgery; HIPEC, hyperthermic intraperitoneal chemotherapy; IQR, interquartile range; SD, standard deviation.

**Table 2:** Biomarker assessment.

Biomarker	Total (*n* = 40)	Adenocarcinoma (*n* = 19)	Mucinous Adenocarcinoma (*n* = 18)
*KRAS* mutation, *n* (%)	(tested = 35)	(tested = 17)	(tested = 16)
G12D	10 (28.6)	5 (29.4)	5 (31.3)
G12V	6 (17.1)	3 (17.6)	3 (18.8)
G12A	5 (14.3) *	2 (11.8)	2 (12.5)
G13H	1 (2.9)	0 (0)	1 (6.3)
G61L	1 (2.9)	1 (5.9)	1 (6.3)
Q61H	1 (2.9)	1 (5.9)	1 (6.3)
No mutation	11 (31.4) **	5 (29.4)	5 (31.3)
*NRAS* mutation, *n* (%)	(tested = 35)	(tested = 17)	(tested = 16)
Q61R	1 (2.9)	1 (5.9)	0 (0)
No mutation	34 (97.1) ***	16 (94.1)	16 (100.0)
*BRAF* mutation, *n* (%)	(tested = 35)	(tested = 17)	(tested = 16)
No mutation	35 (100.0) ***	17 (100.0)	16 (100.0)
MMR, n (%)	(tested = 35)	(tested = 17)	(tested = 16)
Proficient	35 (100.0) ***	17 (100.0)	16 (100.0)
Deficient	0 (0)	0 (0)	0 (0)
HER2 amplification, *n* (%)	(tested = 13)	(tested = 4)	(tested = 9)
Negative	13 (100.0)	4 (100.0)	9 (100.0)
HER2-low	7 (53.8)	3 (75.0)	4 (44.4)
Positive	0 (0)	0 (0)	0 (0)

* Includes one patient with high-grade mucinous appendiceal neoplasm. ** Includes one patient with goblet cell adenocarcinoma. *** Includes one patient with high-grade mucinous appendiceal neoplasm and one with goblet cell adenocarcinoma. HER2, human epidermal growth factor receptor 2; MMR, mismatch repair.

**Table 3:** Treatment information.

Treatment Information	
Number of Lines of Treatment, *n* (%)	
1	40 (100.0)
2	22 (55.0)
3	12 (30.0)
4	3 (7.5)
5	2 (5.0)
First-line treatment, *n* (%)	40 (100.0)
Chemotherapy, *n* (%)	
FOLFOX	21 (52.5)
FOLFIRI	10 (25.0)
FOLFOXIRI	2 (5.0)
CAPOX	2 (5.0)
Capecitabine	3 (7.5)
5-FU	2 (5.0)
Biologics, *n* (%)	
Bevacizumab	20 (50.0)
Dose reduction, *n* (%)	
Yes	17 (42.5)
No	23 (57.5)
Median number of cycles (IQR)	8.50 (5.75, 12.00)
Discontinuation of first-line treatment, *n* (%)	
Disease progression	20 (50.0)
CRS/HIPEC	6 (15.0)
Completed 6 months and planned treatment break	6 (15.0)
Patient preference	3 (7.5)
Decline in ECOG PS	3 (7.5)
Death	1 (2.5)
Ongoing treatment	1 (1.5)
Second-line treatment, *n* (%)	22 (55.0)
Chemotherapy, *n* (%)	
FOLFOX	4 (18.2)
FOLFIRI	14 (63.6)
CAPOX	1 (4.6)
Clinical trials	3 (13.6)
Biologics, *n* (%)	
Bevacizumab	6 (27.3)
Cetuximab	3 (13.6)

FOLFOX, 5-fluorouracil plus leucovorin and oxaliplatin; FOLFIRI, 5-fluorouracil plus leucovorin and irinotecan; FOLFOXIRI, 5-fluorouracil plus leucovorin, oxaliplatin, and irinotecan; CAPOX, capecitabine plus oxaliplatin; 5-FU, 5-fluorouracil; IQR, interquartile range; CRS, cytoreductive surgery; HIPEC, hyperthermic intraperitoneal chemotherapy; ECOG PS, Eastern Cooperative Oncology Group performance status.

**Table 4:** Response rates.

	Doublet Regimens (*n* = 33)	Single-Agent Regimens (*n* = 5)	Triplet Regimens (*n* = 2)
Complete response, *n* (%)	0 (0)	0 (0)	0 (0)
Partial response, *n* (%)	13 (39.4)	0 (0)	0 (0)
Stable disease, *n* (%)	11 (33.3)	3 (60.0)	1 (50.0)
Progressive disease, *n* (%)	7 (21.2)	1 (20.0)	1 (50.0)
Not performed, *n* (%)	2 (6.1)	1 (20.0)	0 (0)

**Table 5:** Prognostic factors for OS.

Factors	Univariate Analysis		Multivariate Analysis	
	HR (95% CI)	*p*-Value	HR (95% CI)	*p*-Value
Sex: Male (vs. female)	2.02 (0.95, 4.28)	0.067	2.83 (1.05, 7.61)	0.039
Age ≥ 65 years	0.89 (0.43, 1.87)	0.764	-	-
BMI (kg/m^2^)				
18.5–24.9	Ref	-	Ref	-
<18.5	4.37 (0.87, 22.09)	0.074	5.25 (0.93, 29.59)	0.06
≥25.0	1.63 (0.71, 3.74)	0.247	2.87 (1.05, 7.87)	0.04
ECOG PS			-	-
0	Ref			
1	1.5 (0.73, 3.09)	0.269		
2	2.71 (0.35, 21.1)	0.342		
Albumin ≥ 3.5 g/dL	0.81 (0.39, 1.68)	0.569	-	-
CEA ≥ 5 U/mL	2.19 (1.03, 4.65)	0.042	2.80 (1.20, 6.52)	0.017
CA19-9 ≥ 37 U/mL	1.52 (0.65, 3.55)	0.329	-	-
Histology			-	-
Intestinal-type adenocarcinoma	Ref			
Mucinous adenocarcinoma	0.86 (0.42, 1.78)	0.686		
Goblet cell adenocarcinoma	1.1 (0.14, 8.5)	0.93		
HAMN	0.61 (0.08, 4.68)	0.634		
Poor differentiation (vs. moderate)	1.68 (0.82, 3.45)	0.154	-	-
Signet ring cell feature	1.24 (0.59, 2.6)	0.563	-	-
*KRAS* mutation	0.68 (0.30, 1.54)	0.353	-	-
FDG PET scan (avid vs. non-avid)	2.02 (0.81, 5.01)	0.13	2.44 (0.94, 6.32)	0.067
Synchronous metastasis (vs. metachronous)	0.61 (0.29, 1.25)	0.175	-	-
Number of metastatic sites (1 vs. ≥2)	1.04 (0.5, 2.18)	0.909	-	-
First-line chemotherapy			-	-
Doublet regimen	Ref			
Single-agent regimen	1.22 (0.46, 3.26)	0.689		
Triplet regimen	1.67 (0.39, 7.21)	0.492		
Addition of bevacizumab	1.16 (0.58, 2.34)	0.674	-	-
Prior CRS/HIPEC	0.65 (0.25, 1.69)	0.373	-	-

BMI, body mass index; CA19-9, carbohydrate antigen 19-9; CEA, carcinoembryonic antigen; CI, confidence interval; CRS, cytoreductive surgery; ECOG PS, Eastern Cooperative Oncology Group performance status; FDG, fluorodeoxyglucose; HAMN, high-grade appendiceal mucinous neoplasm; HIPEC, hyperthermic intraperitoneal chemotherapy; HR, hazard ratio; OS, overall survival; PET, positron emission tomography; Ref, reference.

## Data Availability

The datasets used and analyzed in the current study are available from the corresponding author upon reasonable request.

## Supplementary Materials

The following supporting information can be downloaded at: https://www.mdpi.com/article/10.3390/cancers17203297/s1, Table S1: Subsequent treatments information; Table S2: Response rates in patients who received first-line doublet chemotherapy; Table S3: Response rates in patients who received first-line doublet chemotherapy with and without bevacizumab; Table S4: Response rates for adenocarcinoma and mucinous adenocarcinoma subtypes.

## Abbreviations

The following abbreviations are used in this manuscript:

5-FU	5-fluorouracil
CA19-9	carbohydrate antigen 19-9
CAPOX	capecitabine plus oxaliplatin
CEA	carcinoembryonic antigen
CI	confidence interval
CR	complete response
CRC	colorectal cancer
CRS	cytoreductive surgery
ECOG	Eastern Cooperative Oncology Group
EGFR	epidermal growth factor receptor
FDG	fluorodeoxyglucose
FOLFIRI	5-FU plus leucovorin and irinotecan
FOLFOX	5-FU plus leucovorin and oxaliplatin
FOLFOXIRI	5-FU plus leucovorin, oxaliplatin, and irinotecan
HER2	human epidermal growth factor receptor 2
HIPEC	hyperthermic intraperitoneal chemotherapy
HR	hazard ratio
MMR	mismatch repair
ORR	objective response rate
OS	overall survival
PD	progressive disease
PET	positron emission tomography
PFS	progression-free survival
PR	partial response
SD	stable disease
