# Supplementary material for: Survival Outcomes and Prognostic Factors in Metastatic Unresectable Appendiceal Adenocarcinoma Treated with Palliative Systemic Chemotherapy: A 10-Year Retrospective Analysis from Australia
Source: Cancers (Basel). 2025 Oct 11;17(20):3297. doi: 10.3390/cancers17203297 (PMC12562899; doi:10.3390/cancers17203297)
Supplement: Supplementary file 1 [file cancers-17-03297-s001.zip › cancers-3876494-supplementary.pdf]

Supplementary Materials

Table S1. Subsequent treatments information

First-line treatment, n (%)	40 (100.0)
**Regimens, n**	
FOLFOX	12
FOLFOX/bevacizumab	9
CAPOX/bevacizumab	2
FOLFIRI	5
FOLFIRI/bevacizumab	5
FOLFOXIRI	1
FOLFOXIRI/bevacizumab	1
Capecitabine/bevacizumab	3
5-FU	2
Second-line treatment, n (%)	22 (55.0)
**Regimens, n**	
FOLFOX	3
FOLFOX/bevacizumab	1
CAPOX/bevacizumab	1
FOLFIRI	7
FOLFIRI/bevacizumab	4
FOLFIRI/cetuximab	3
Clinical trials	3
**Median number of cycles** (IQR)	8.00 (4.25,12.00)
**Discontinuation, n**	
Disease progression	14
CRS/HIPEC	3
Patient preference	2
Death	2
Decrease in ECOG PS	1
Third-line treatment, n (%)	12 (27.5)
**Regimens, n**	
FOLFOX	2
FOLFOX/bevacizumab	1
FOLFOX/cetuximab	1
FOLFIRI	3
FOLFIRI/bevacizumab	1
FOLFIRI/cetuximab	1

TAS102	1
Irinotecan/cetuximab	1
Clinical trials	1
Fourth-line treatment, n (%)	3 (7.5)
**Regimens, n**	
FOLFOX	1
TAS102	1
Clinical trials	1
Fifth-line treatment, n (%)	2 (5.0)
**Regimens, n**	
TAS102/bevacizumab	2

5-FU, 5-fluorouracil; CAPOX, capecitabine plus oxaliplatin; CRS, cytoreductive surgery; ECOG PS, Eastern Cooperative Oncology Group performance status; FOLFIRI, 5-fluorouracil plus leucovorin and irinotecan; FOLFOX, 5-fluorouracil plus leucovorin and oxaliplatin; FOLFOXIRI, 5-fluorouracil plus leucovorin, oxaliplatin, and irinotecan; HIPEC, hyperthermic intraperitoneal chemotherapy; IQR, interquartile range; TAS102, trifluridine-tipiracil.

Table S2. Response rates in patients who received first-line doublet chemotherapy.

	Oxaliplatin-based (n=23)	Irinotecan-based (n=10)
Complete response, n (%)	0 (0)	0 (0)
Partial response, n (%)	10 (43.5)	3 (30.0)
Stable disease, n (%)	7 (30.4)	4 (40.0)
Progressive disease, n (%)	4 (17.4)	3 (30.0)
Not performed, n (%)	2 (8.7)	0 (0)
Objective response rate, n (%)	10 (43.5)	3 (30.0)

Table S3. Response rates in patients who received first-line doublet chemotherapy with and without bevacizumab.

	Bevacizumab (n=17)	Without bevacizumab (n=16)
Complete response, n (%)	0 (0)	0 (0)
Partial response, n (%)	7 (41.2)	6 (37.5)
Stable disease, n (%)	6 (35.3)	5 (31.2)
Progressive disease, n (%)	2 (11.7)	5 (31.2)
Not performed, n (%)	2 (11.7)	0 (0)
Objective response rate, n (%)	7 (41.2)	6 (37.5)

Table S4. Response rates for adenocarcinoma and mucinous adenocarcinoma subtypes.

	Adenocarcinoma (n=19)	Mucinous adenocarcinoma (n=18)
Complete response, n (%)	0 (0)	0 (0)
Partial response, n (%)	7 (36.8)	4 (22.2)
Stable disease, n (%)	5 (26.3)	10 (55.6)
Progressive disease, n (%)	4 (21.1)	4 (22.2)
Not performed, n (%)	3 (15.8)	0 (0)
Objective response rate, n (%)	7 (36.8)	4 (22.2)
